# Home based dialysis systems in developing countries

**DOI:** 10.7189/jgh.10.010312

**Published:** 2020-06

**Authors:** Hassan Ahmed, Syed Inam Ur Rehman

**Affiliations:** ^1^Dow University of Health Sciences, Karachi, Pakistan

With the increasing life expectancy, thanks to better than ever health care comes an increased burden of chronic diseases. One such illness causing significant mortality and morbidity is End-Stage Renal Disease (ESRD). ESRD mainly warranting dialysis, has a prevalence rate of 8%-16% [1] worldwide. With the increment in cases of chronic renal disease over the span of past years and overwhelmed state of health care borne witness in the developing world, we confront a growing need than ever of having Home-based dialysis treatments brought into the mainstream.

Conventional hemodialysis (HD) is typically performed thrice a week in a 4-hour per session regime (12 hours a week). Apart from how much time taking this regimen is, taking a big chunk out of patient’s week, it carries a 7-fold increased risk of mortality when compared to the general population [2]. Needless to say, the routine treatment leads to an array of physical and emotional disturbance which when met with economic burden further demeans the quality of life. Overstressed and overworked individuals, in turn, impede the progress of a state en route to success.

Home-based dialysis systems aim to deal with a multitude of such concerns and relieve the users of a nuisance. The effectiveness and outcomes of Home-based dialysis make it a very viable option for consideration in our opinion. A multitude of home-based options are available like peritoneal dialysis, daily home HD, nocturnal home HD, standard home HD etc. An appropriate option can be selected after considering a patient’s needs, comfort and practicality.

First of all, this system provides credible results boasting decreased mortality that could be ascribed to a more intensive dialysis regime (>12 hours) enabled by the flexibility of schedule at home [3]. It additionally allows patients to perform HD nocturnally for a good 8 hours. Such high doses result in fewer fluctuations in the internal milieu of the patient. Multiple randomized controlled trials (RCTs) of nocturnal hemodialysis are reported to have linked intensive HD regimes with improvements in the left ventricular mass index and left ventricular hypertrophy [4]; both factors having been accredited to increased mortality in ESRD and dialysis patients. Such intensive regimes are implausible in institutional settings hence further supporting the application of home HD.

The ordeal of thrice a week dialysis, metabolic derangement in those with ESRD, and vocational and psychosocial effects of dialysis dependence are likely attributable to the decreased health-related quality of life (HRQOL). The convenience of home HD that can be customized as per one’s schedule or performed nocturnally contributes to a decrease in derangements yielding enhanced productivity at the workplace and a profoundly raised HRQOL [5]. For example, nocturnal HD is ideal for people who have a day time job and can’t attend dialysis sessions at a hospital while standard home HD is more convenient for stay at home patients who like this recurring task to be performed in the comfort of their homes. Individuals performing dialysis at their homes feel much more in control of their treatment and are have significantly smaller stress. This positive state of mind helps them in their professional life to do a much better job than their Conventional HD counterparts.

**Figure:**
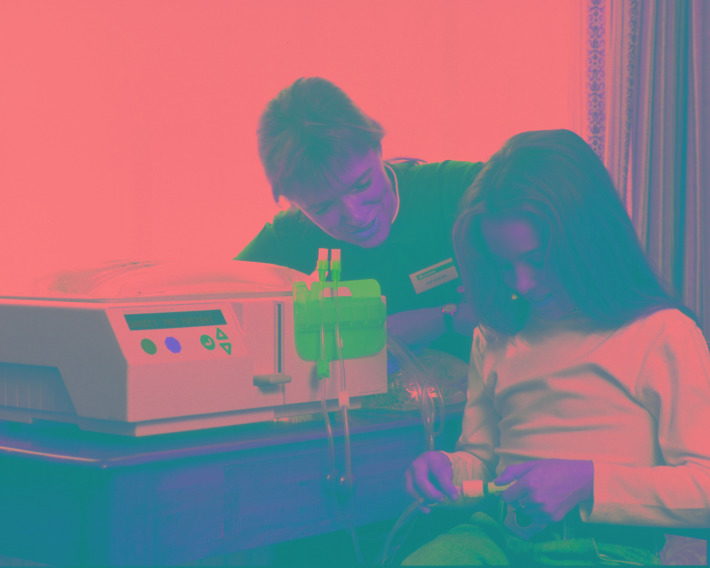
Photo: Health professional helping patient with peritoneal dialysis (Source: Wikimedia Commons, licensed under the Creative Commons Attribution-Share Alike 3.0 Unported licence).

These are generally cost-effective as compared to conventional HD [5] – home HD decreases expenditure by the lower requirement of skilled personnel, lesser traveling expenses, and decreased absence from work. While there is an increased cost initially to train the patients and their caregivers, then making all the necessary equipment and supplies available at patients home but in the longer run, due to the decreased need for skilled staff, better resource utilization and far lesser complications, a health care system is expected to save a lot from their already small budget. This combined with the ability of these patients to join the workforce means they can now contribute to the country’s economy. These factors are imperative for nations that can’t bear to lose manpower and aim to ease the financial burden of citizens.

Although home-based dialysis is a great option for patients, the supporting framework and trained health care providers for it is not readily available, especially in the developing countries. Healthcare care providers need adequate training and confidence so they can guide and counsel their patients properly. Patients and their caregivers need to be trained usually for 4-6 weeks to safely administer dialysis at home. Training is usually achieved by means of short courses and instructional videos. Some patients may require a nurse to visit them during their sessions. Modifications like plumbing and wiring may need to be done to adjust the equipment inside the patient’s house. To make this all possible, strong determination from the authorities is very much essential. But in the long run, it helps both the patients and health care systems tremendously. That’s the reason a number of countries are looking into the “home dialysis first” policy.

Safe to say, implementing a home-based dialysis model will render undeniable assistance to patients subjected to growing global economies. A great majority of patients are unaware of the availability of Home HD as a treatment option. Patients should be informed that besides their conventional in-center HD, Home-based HD can also be an option for them. All the benefits and pitfalls should be properly explained so they can take an informed decision. Governments and health care bodies are encouraged to look into Home HD for their population. Existing medical facilities should be upgraded to support home HD. Medical personnel and nephrologists should be trained in the discipline of home HD to provide the care remotely. Appropriate patient and caregiver education should be carried out and patients should be carefully selected and monitored to make the endeavor successful.
